# Production of Composite Briquette Fuel from Brewery Wastewater Sludge and Spent Grains

**DOI:** 10.1155/2024/1710628

**Published:** 2024-09-21

**Authors:** Bontu Teshome, Berhanu Assefa, Kenatu Angassa

**Affiliations:** ^1^Chemical and BioEngineering Department, Addis Ababa University, P.O. Box 1176, Addis Ababa, Ethiopia; ^2^Department of Environmental Engineering, Addis Ababa Science and Technology University, P.O. Box 16417, Addis Ababa, Ethiopia; ^3^Sustainable Energy Center of Excellence, Addis Ababa Science and Technology University, P.O. Box 16417, Addis Ababa, Ethiopia

## Abstract

Biomass waste energy recovery is a significant method for recycling energy from waste and capturing it for use in renewable energy sources. The abundance of brewing byproducts, such as brewery spent grain (BSG) and brewery wastewater sludge (BWWS), as well as their high carbon concentrations gives these wastes energy potential. With 20% molasses utilized as a binding agent to maximize the high caloric value of the briquette, this study sought to examine the quality of mixed briquettes made from BSG and BWWS. In order to make composite briquettes with a maximum caloric content of 19.94 MJ/kg, the ideal conditions were chosen, which included a temperature of 350°C, a production period of 60 min, and a 75% BSG mixing ratio. It can be compared to sawdust briquettes, which have a calorific value of 22.88 MJ/kg, by looking at the calorific value of densified with pressure 100 bar for mixed carbonized briquettes vs mixed noncarbonized briquettes (21.13 MJ/kg). The value of *R*^2^ was 0.9607 and indicated that 96.07% of the total validation in the calorific value correlation between experimental and predicted values. The finding of the study showed that the efficiency of the quadratic model in fitting the data would be higher under the conditions of the experiment. Based on ISO 17225-6 fuel quality classes and specifications for graded nonwoody pellets, the study concluded that using BSG and BWWS as alternative energy sources meets those requirements.

## 1. Introduction

Due to the world's fast growing industrialization and urbanization, industrial solid waste pollution has become a problem that affects everyone on the planet [1]. Nevertheless, as was previously indicated, industrialization and urbanization have also had significant negative effects on the environment and human health [2]. Environmental pollution is the primary issue associated with the fast industrialization, urbanization, and improvement of people's living standards. This is due to inadequate technology for the treatment, recycling, and disposal of industrial solid wastes.

Due to the fact that emerging businesses produce significant amounts of industrial solid waste each year, developing nations have been having issues. Although the brewing industry generates a lot of waste, it is one of the most important industries for the economic growth of a nation. Controlling these brewery solid wastes is essential for reducing environmental contamination through effective treatment, reuse, recycling, and discharge. However, many developing nations lack the necessary treatment, recycling, and disposal technology. These wastes can be dealt with practically by reducing trash, recycling waste, and reusing materials [3].

The majority of breweries use biological treatment technologies to handle their wastewater, which results in a lot of sludge being produced from brewery wastewater (BWWS). The biological treatment of brewery wastewater results in the production of (BWWS). As a result, the brewery's BWWS production is growing along with the year-over-year increase in beer production. The brewery is now disposing of BWWS by applying it to agricultural areas and landfills, which has a negative influence on the environment and the environment's surface and groundwater supplies.

However, there are significant issues for the brewery and the environment related to the expensive expense of landfilling, the lack of available land, as well as public health issues brought on by the disagreeable stench. Another brewery by-product, brewery spent grain (BSG), has primarily been fed to dairy cattle as a feed. In terms of total solid waste produced throughout the brewing process, it accounts for around 85% of the total. The production of 14 to 20 kg of BSG per hectoliter of beer is estimated. [4].

Due to their high levels of organic matter with an acceptable high heating value, spent grains and brewery wastewater sludge can be used as a possible resource for the production of renewable fuel briquettes [5]. With this approach, waste transportation expenses will be decreased, landfilling expenses will be decreased, landfill lifespans will be increased, trash will be converted into energy, and the effects on the environment and human health will be reduced [6]. In order to generate refuse-derived fuel briquettes for various purposes, many studies have been conducted using biomass and industrial wastewater sludge [7].

Ethiopia has to explore alternative energy sources in order to achieve sustainable economic growth, given its current state of energy dependency. Consequently, converting organic wastes into fuel briquettes, such as spent grains and sludge from breweries, is a viable substitute for more costly and environmentally friendly domestic energy sources. It also creates jobs and enhances the economy [8]. The treatment, recycling, and energy recovery of these brewery solid wastes may be accomplished by the production of fuel briquettes [9]. If their moisture level is kept below 14%, sludge-based briquette fuels have a calorific value that is comparable to fuel coal, ranging from 17 to 25 MJ/kg. As a fuel briquette, this qualifies it as a suitable source of energy for homes [9].

Several researchers investigated the production of briquettes from a variety of agricultural wastes, including sugarcane trash dry leaves, coffee husk, grass [10], *Oxytenanthera abyssinica*, *Prosopis juliflora*, *Arundinaria alpina, and Acacia melifera* [11], khat waste [12], sawdust and caw dung admixture [13], coffee husk [14], and sawdust and brewery wastewater sludge [15]. The study conducted from brewery spent grains and spent coffee grounds showed that the carbon content was found in the SCG (51.44%) which was 12% higher than that found in BSG, whereas the oxygen content of BSG (45.35%) was higher than that observed for SCG, which was 39.07% [16]. Brewery wastewater sludge and spent grain combined to produce production briquettes, although this research has not been published. According to the study, combining spent grain with brewery wastewater sludge was result in high-quality mixed briquettes that contribute to both a more sustainable approach to waste management as well as the energy mix. As a result, the objective of this study is to investigate briquette fuel made from used spent grains and brewery wastewater sludge utilizing molasses as a binder.

## 2. Methods

### 2.1. Sample Preparation

Molasses was gathered from the National Alcohol and Liquor plant in Addis Ababa, while spent grain and brewery effluent sludge were collected from Heineken Kilinto Brewery. The samples were taken right at the factory's discharge site. Between December 2020 and July 2021, experimental work was conducted [17]. The samples were dried so that the moisture level was fewer than 15%. The American Society of Testing Materials' standards were used to evaluate the samples' moisture content (ASTM D3173-03, 2004).

The samples were subsequently reduced in size using an attrition mill to fit the specifications of the characterizations. Using a muffle furnace, the BWWS and BSG were carbonized for one to two hours at a temperature between 350 and 450°C. The samples were then ground up and combined in proportions of 25%, 50%, and 75% brewery wasted grain (BSG) and brewery wastewater sludge (BWWS). The mixture of carbonized, crushed, and blended spent grains with brewery wastewater sludge was then combined with 20% molasses as a binder. The Ethiopian Geological Survey's laboratory determined the calorific value while the Addis Ababa Institute of Technology's (AAiT) School of Chemical and Bioengineering laboratory carried out the carbonization and briquetting processes. At the Addis Ababa College of Natural Science (AACNS), ultimate analysis was carried out to ascertain the elemental composition of the materials.

### 2.2. Characterization of Samples

#### 2.2.1. The Proximate Analysis

According to the American Society of Testing Materials, the BWWS and BSG's moisture, volatile matter, ash, and fixed carbon contents were measured. Moisture content (MC) was determined using the method (ASTM D3173-03, 2004) and calculated from equation (1). Ash content was determined using the ASTM method (ASTM D3174-02, 2004) and calculated from equation (2). Volatile content was determined by the ASTM method (ASTM D3175-02, 2004) and calculated from equation (3). Fixed carbon content is a calculated value, and it is the resultant of the summation of percentage moisture, ash content, and volatile matter subtracted from 100. All contents shall be taken from the same moisture base and calculated from

(1) $$\begin{array}{c} \%\text{MC}=\left[\frac{W_{o}\;-\;W_{f}}{W_{o}}\right]\times 100, \end{array}$$

where *W*_*o*_ = initial weight of the sample before drying and *W*_*f*_ = final weight of the sample after drying.

(2) $$\begin{array}{c} \%\text{AC}=\left[\frac{A-B}{C}\right]\times 100, \end{array}$$

where *A* = weight of crucible, cover, and ash residue, g, *B* = weight of empty crucible, and cover, g, *C* = weight of the sample used, g.

(3) $$\begin{array}{c} \%\text{VM}=C-D, \end{array}$$

where *A* = weight of sample used, g. *B* = weight of the sample after heating, g. *C* = weight loss, %. *D* = moisture content %.

(4) $$\begin{array}{c} \text{Fixed Carbon}\%=100–\left(\text{MC }\left(\%\right)+\text{AC }\left(\%\right)+\text{VM }\left(\%\right)\right). \end{array}$$

#### 2.2.2. Ultimate Analysis

To determine the concentrations of key chemical constituents in the biomass, a comprehensive investigation of its chemical composition was conducted. It was determined at Addis Ababa University using an elemental analyzer (EA 1112 Flash CHNS/O- analyzer) and the ASTM techniques. At the Ethiopian Geological Survey, an adiabatic bomb calorimeter was used to calculate the calorific values of each sample.

### 2.3. Experimental Design

The experiment was designed with the Central Composite Design Method (CCD) through response surface methodology (RSM) using Design-Expert version 12 software. The experiments were carried out with three factors (temperature, time, and mixing ratio) at three levels (minimum, center point, and maximum) controlled to see their effect on calorific value as presented in Table 1. Response surface methodology (RSM) is an empirical modelling strategy for determining the correlations between various operation factors and response variables. The primary goal of employing RSM is to optimise these variables while keeping the intended value of the response function in mind. The interaction effects of independent variables and their influences on response were investigated, and the factors were optimised.

### 2.4. Briquettes Production

The optimal carbonized sample was pulverised and sieved with less than 1 mm mesh size before being weighted and blended with each mixing ratio. The mixed optimal carbonized samples were then well homogenised and mixed with 20% molasses as a binding agent. Similarly, noncarbonized samples and sawdust samples were thoroughly homogenised after being mixed with 20% molasses as a binding agent.

Then, using a press at 1 bar densifier (hydraulic press), each measured mixture sample was placed into a cylindrical form mould with a diameter of 42 mm and a length of 52 mm. According to [18], the hydraulic press was primarily employed to compact both carbonized and noncarbonized briquettes. The briquette sample was removed from the cylindrical mould after being manually squeezed and waited for 15 minutes. Finally, the briquettes were sun-dried for 3 to 4 days before being stored in a covered container to minimize moisture absorption from the surrounding humidity [19].

### 2.5. Briquettes Characterization

Finally, the optimal briquette was analysed using ASTM standards [20] to evaluate its physical and chemical parameters such as moisture content, volatile matter content, ash content, fixed carbon content, density, and heating value (HV).

### 2.6. Data Analysis

SPSS software (Version 20) was used for statistical analysis to compare the carbonized briquettes of BSG and BWWS, noncarbonized BSG and BWWS, and sawdust briquettes using a one-way Analysis of Variance (ANOVA) that was performed at a 95% significant level (*P* < 0.05).

## 3. Results and Discussion

### 3.1. Raw Material Characterization

Considering the energy profile, moisture content, and calorific value (HHV) of biomass is important when deciding whether to use it for energy. An elemental/ultimate, proximate, and calorimetric analysis was performed for this characterization.

#### 3.1.1. Proximate Analysis

Table 2 shows the moisture content, volatile matter content, ash content, fixed carbon content, and calorific value of BSG, BWWS, and sawdust.

*(1) Moisture Content*. At the factory's disposal point, BSG and BWWS had significant moisture contents of 71% and 88%, respectively, making them challenging to store and discard. Using biomass waste materials has certain disadvantages, one of which is its high moisture content. Biomass moisture is an aqueous solution that has been mineralized and contains neutral species, cations, and anions [21]. In order to lower the energy required for carbonization, Asamoah et al. [9] state that the biomasses must be dried to a moisture content of less than 15% before carbonization. To lower the moisture level below 15%, the samples were sun-dried for four days. The moisture of the BWS was then lowered to 5.11% and that of the BWWS to 5.83%. As long as the sawdust's moisture level was 6.93%, the carbonization process could have taken place. As a study conducted on blending, the brewery spent grain (BSG) and spent coffee ground (SCG) into biomass composite were showed that reduced the moisture content by 10–15% wt [21]. Determining the moisture content of the raw materials is crucial to the biomass briquetting process [22]. By expanding the contact area between particles, which is necessary to increase their cohesion strength, water acts as a binder and aids in the formation of hydrogen bonds and Vander Waals forces.

*(2) Volatile Matter*. The volatile matter of the BSG, BWWS, and sawdust, as shown in Table 2, was within the permissible range of 50–90%, which satisfies the criteria for briquettes outlined by numerous researchers [23]. The best quality briquette is produced when greater volatile and lower volatile materials are blended [24]. Inflammable or inflammable gases, or a combination of the two, emitted during burning make up volatile matter, which is a mixture of short- and long-chain hydrocarbons. The burning behavior of briquettes is significantly impacted by these gases [25]. Lower volatile matter is a sign that the briquettes may be difficult to ignite, but once they are, they burn smoothly, whereas higher volatile matter produces good combustibility at low ash content [26].

*(3) Ash Content*. For greater fuel quality, a low ash percentage is necessary and advantageous since it increases heating value, reduces fouling and slagging, and inhibits corrosion [21]. The ash levels of the BSG, BWWS, and sawdust were found to be 4.92%, 20%, and 1.90%, respectively. The level of ash of the BSG and sawdust was almost at the permissible values; however, the BWWS had a slightly higher percentage of ash. This paradox can be greatly mitigated by combining the BSG and BWWS. Brewery sludge needs to be mixed with biomass waste because of its high ash level, which makes it less combustible and has a poor calorific value [15]. The lower the ash level, the higher the quality of the briquette. For fuel briquettes, an ash percentage tolerance of roughly 4% is acceptable [27]. Moreover, the low ash content suggests a high specific heat of combustion or heating value [28]. On the other hand, a higher ash content in fuel briquettes typically results in larger emissions of dust and other air pollutants, which also impacts the volume and effectiveness of combustion [29]. The ash content of the BSG was ranged from 3.16 to 3.82% [21], which was slightly lower than the present study result.

*(4) Fixed Carbon*. A fixed carbon content of 15.25% was found in sawdust, 3.05% in BSG, and 15.05% in BWWS, as per previous research [28]. The fixed carbon content of BSG and sawdust was within acceptable limits and suitable for briquette manufacture; however, the fixed carbon content value of BWWS was slightly low and could be significantly raised by blending with BSG. Fixed carbon, which serves as the primary heat generator during combustion, provides a rough indication of a fuel's heating value [30]. Better and more efficient fuel briquettes require a lower concentration of ash and volatile materials and a higher fixed carbon content [9]. Briquettes' proportion of fixed carbon content is a significant variable that affects the calorific value of fuel [31].

*(5) Calorific Value*. There were 20.12 MJ/kg, 13.80 MJ/kg, and 20.28 MJ/kg of calorific value for the BSG, BWWS, and sawdust, respectively (Table 2). The calorific values are nearly the same, with the exception of the sludge, whose lower amount of fixed carbon and higher ash content causes it to have a lower calorific value than the others. It can be enhanced by the carbonization process and combining with BSG. The energy content of the fuel is determined by its calorific value. It is a characteristic of biomass fuel that depends on its chemical make-up and level of moisture [32]. There is a clear relationship between the elemental contents and fixed carbon content of biomass-based briquettes and their calorific value.

#### 3.1.2. Ultimate Analysis

Table 3 presents the final BSG, BWWS, and sawdust analyses. The biomass's oxygen content was calculated using the difference between those values and the sum of carbon, nitrogen, hydrogen, and sulphur. These findings demonstrate that the samples analysed contained highly excellent levels of carbon and hydrogen for use as sources of energy for heating homes.

Compared to BSG and sawdust, BWWS had a reduced carbon concentration. Briquettes burn poorly due to the lower carbon content in BWWS [15]. The hydrogen percentage in BSG was likewise higher than that in BWWS, and other research findings indicated that the hydrogen content for all dry biomass was between 5 and 7 percent [33]. BWWS had a somewhat higher nitrogen level than BSG and sawdust. Sulphur test results showed that it was below the equipment' detection threshold. The oxygen concentration of BWWS is higher than that of BSG and sawdust, which lowers the amount of air required for combustion. The outcome was consistent with earlier research into BWWS and sawdust [15, 34, 35]. The oxygen content of the sample was determined by the difference between the sum of carbon, nitrogen, hydrogen, and sulfur and 100. The carbon content of sawdust was higher than the carbon content of BWWS [15]. The oxygen content of BSG (41.8%) is slightly higher than the study result reported from the other study of BSG (39.87%) [21], whereas the carbon content (49.5%) of BSG in present study is slightly lower than the result reported by other study which was 51.2% [21].

### 3.2. Experimental Design

Table 4 lists the results of the 20 experiments that were conducted using the BSG: BWWS mixture at various mixing ratios of 25 : 75, 50 : 50, and 75 : 25. In order to manufacture the composite briquettes with the highest caloric value, the ideal circumstances of 350°C, 60 minutes, and 75% of BSG mixing ratio were chosen.

Carbonization at a lower temperature generates a significant amount of char while achieving the highest calorific value. Since the spent grain contains the higher energy content in the blending process, the time target was kept within bounds and the mixing ratio was maximized. The level of satisfaction of the optimal conditions for the ultimate objective of response, as shown in Table 5, was then assessed using the composite desirability, which has a value between 0 and 1.

### 3.3. Briquette Production

For both carbonized and noncarbonized briquettes made from the mixture of BSG and BWWS as well as briquettes made from sawdust, 20% molasses was employed. Biomass char was briquetted using a hydraulic press with a 50-ton capacity. As shown in Table 6, the bulk density of noncarbonized briquettes with a constant compression pressure of 100 bar was 1.118 g/cm^3^, and it increased to 1.247 g/cm^3^ after the carbonization process.

The calorific value of the best-mixed carbonized sample was 19.95 MJ/kg, while the calorific value of the best-mixed carbonized briquette densified at 100 Bar was 22.55 MJ/kg. This study demonstrates the variations between the noncarbonized, carbonized, and sawdust briquettes in terms of their physical characteristics. The briquetting process' operating factors would have an impact on the briquettes' quality. Densification and the use of molasses as a binder have improved the briquettes' physical and mechanical characteristics. The primary goals of the densification process included improving the calorific value and appropriateness for handling, storage, and transportation [15]. The mixed samples' calorific value was increased by carbonizing them to levels that are on par with those of sawdust briquettes.

### 3.4. Characterization of the Briquette

#### 3.4.1. Proximate Analysis

A percentage weight basis for moisture, ash, and volatile matter is provided by the proximate analysis. The differences between the results of the sawdust briquette, the optimal mixed raw sample (75% BSG to 25% BWWS), and the carbonized mixed sample values can be attributed to the volatilization that occurs during carbonization, which results in an increase in the content of ashes and fixed carbon while a decrease in the content of moisture and volatile matter, as shown in Table 7.

When the raw material has a high moisture content, the heating value and fuel efficiency are decreased. Additionally, the majority of automation systems are unable to respond to sudden changes in moisture content, which causes incomplete combustion and higher emissions. Both the carbonized and noncarbonized briquettes have moisture contents of 5.37 and 2.92, respectively. It is clear from the results that carbonization causes the raw material's water content to drop. Dark smoke, heat loss, pollution risk, and soot buildup on the boiler surface are all effects of complete combustion of volatile substances. The mixed sample that has not been carbonized is more reactive and has more volatile components than the pellet sample that has been carbonized. Biomass burns quickly and is difficult to manage because of the extremely volatile materials it contains. A significant volume of secondary air under high pressure must be delivered at a key area for efficient combustion if the fuel contains a larger percentage of volatile stuff.

#### 3.4.2. Analysis of Variance (ANOVA) of the Model

Design expert version 12 was used to analyse the interactions between each variable and its impacts on the calorific value for each variable process (temperature (*A*), time (*B*), and mixing ratio (*C*)). The response surface was identified as a quadratic model by the ANOVA analysis results. Measured by the Model *F*-value, the ANOVA model as a whole is significant. A substantial model is implied by the model's Model *F*-value of 27.15. An *F*-value this large being caused by noise only happens with a 0.01% chance. Model terms are significant when their *P* values are lower than 0.0500. Here, important model terms include *A*, AB, AC, BC, and *B*^2^. Indicators of the model terms' significance are values greater than 0.1000. Consequently, the terms *B*, *C*, *A*^2^, and *C*^2^ are not important model terms. Model reduction might help your model if it has a lot of meaningless terms (except those needed to sustain the hierarchy). It is crucial to ensure that the model fits in order to provide a good approximation of reality; otherwise, the outcomes of optimization could produce incorrect findings and conclusions. The lack of fit *F*-value of 0.41 indicates that it is not significant in comparison to the pure error. When the lack of fit *F*-value is this high, noise has an 82.26% likelihood of being the cause. The model should fit since a nonsignificant lack of fit is desirable.

The R-squared (*R*^2^), which measures the proportion of caloric value change that can be accounted for by variation in the explanatory (independent variable), was used to assess the fit quality of the statistical model (Table 8). The validation of the statistical model depended heavily on it. The calorific value connection between experimental and predicted values and the independent factors can account for 96.07% of the variation in the dependent variable, according to the validation result for equation (6), which showed an *R*^2^ value of 0.9607. The quadratic model would fit the data more effectively under the experimental conditions the greater the value of *R*^2^.

The adjusted *R*^2^ of 0.9253 and the predicted *R*^2^ of 0.8976 are reasonably congruent. This demonstrated that the observed and expected values agreed fairly well. The discrepancy between adjusted *R*^2^ and predicted *R*^2^ is less than 0.2, which is acceptable. Another model fit statistics number that gauges signal-to-noise is “Adeq Precision.” It displays the range of variation in the anticipated dependent variable to an estimate of standard error. It was obtained by subtracting the least expected value from the maximum and dividing the result by the predicted value's average standard deviation. A ratio of at least 4 is preferred. An appropriate signal is indicated by the analysis ratio of 21.283. The model can therefore be used to explore the design space.

The coefficient of variance (CV%) is a measurement of how much of the experimental data's residual variation is in relation to the size of the mean. A higher coefficient of variance numbers indicates that the experiment is not very reliable. It was discovered in this experiment that the coefficient of variance was 4.22, which was less, and that the experiment was trustworthy and had good precision. The independent variable experimental values and the dependent variable actual values (response) from 20 experimental runs were used to predict the equations of the model. The experimental data had a quadratic equation fit that, according to the ANOVA, was the best. Every independent variable's importance to the dependent variable was demonstrated using the quadratic model. Equation (5) gives the quadratic model equation for the carbonization of mixed BSG and BWWS, while equation (6) gives the equation in terms of actual components. One-factor coefficients demonstrate the impact of a single variable, while two-factor coefficients suggest the impact of two variables working together. Coefficients with second-order factors similarly demonstrated the quadratic impact of the factors.

(5) $$\begin{array}{c} \text{Caloric Value}=+3407.1-395.1A+71.8B+82.9C-183.6\text{AB}-323.5\text{AC}-486.9\text{BC}-26.9A^{2}+501.8B^{2}-154.3C^{2}, \end{array}$$

(6) $$\begin{array}{c} \text{Caloric Value}=-4137.8+24.7A-16.5B+190.0C-0.12\text{AB}-0.26\text{AC}-0.65\text{BC}-0.01A^{2}+0.56B^{2}-0.25C^{2}. \end{array}$$

According to the quadratic equation model in equation (5), the calorific value was positively impacted by time and mixing ratio, while the response was negatively impacted by temperature. With a strong linear effect on calorific value, temperature has a negative regression coefficient of −395.1, but time and mixing ratio have positive regression coefficients of 71.8 and 82.9, respectively. All three linear terms (*A*, *B*, and *C*), quadratic terms (*A*^2^, *B*^2^, *C*^2^), and interaction quadratic terms (AB, AC, and BC) were used to define the calorific value. The response (calorific value) was positively impacted by the factors with positive coefficients for the linear terms of *B* and *C* and the quadratic term *B*^2^, but the response was negatively impacted by the factors with negative coefficients for the linear term *A*, interaction terms AB, AC, and BC, as well as the quadratic terms *A*^2^ and *C*^2^.

#### 3.4.3. Diagnostics Plots

**Figure 1:**
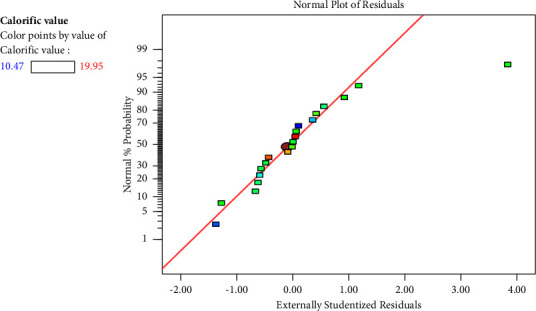
Normal probability plot.

**Figure 2:**
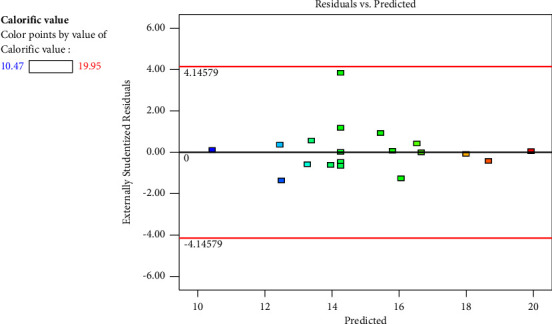
Studentized versus residual plots.

In Figure 1, the normal probability graph is displayed. The plot of internal studentized residuals versus normal probability appears to virtually follow a linear straight line, indicating that the mistakes were normally distributed. The residuals' normal distribution can be determined by looking at the normal probability. Since the residuals in this instance practically formed a straight line, the error distribution must be normal. Only one point deviated from the straight line, as shown in Figure 1, although such situations are common. The residuals were plotted against the rising projected values of the dependent variable (response) in Figure 2. It was dispersed at random and had a constant variance throughout the graph.

**Figure 3:**
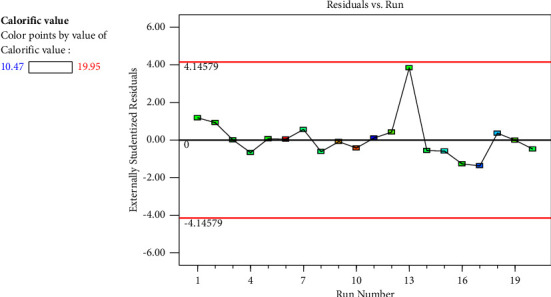
Residuals versus run plot.

**Figure 4:**
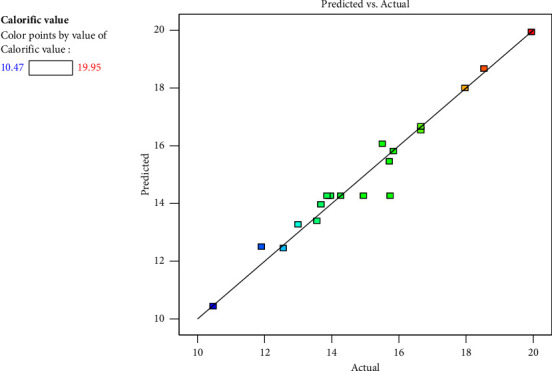
Predicted versus actual plot.

Figure 3 depicts the residual vs run plot, which showed how the experimental run was randomised.  Figure 4 displays a plot of the derived predicted values vs the dependent variable's actual values. The results in the picture showed that the quadratic model's regression equation provided a very accurate representation of the experimental data. Near the linear straight line, all of the points are located.

**Figure 5:**
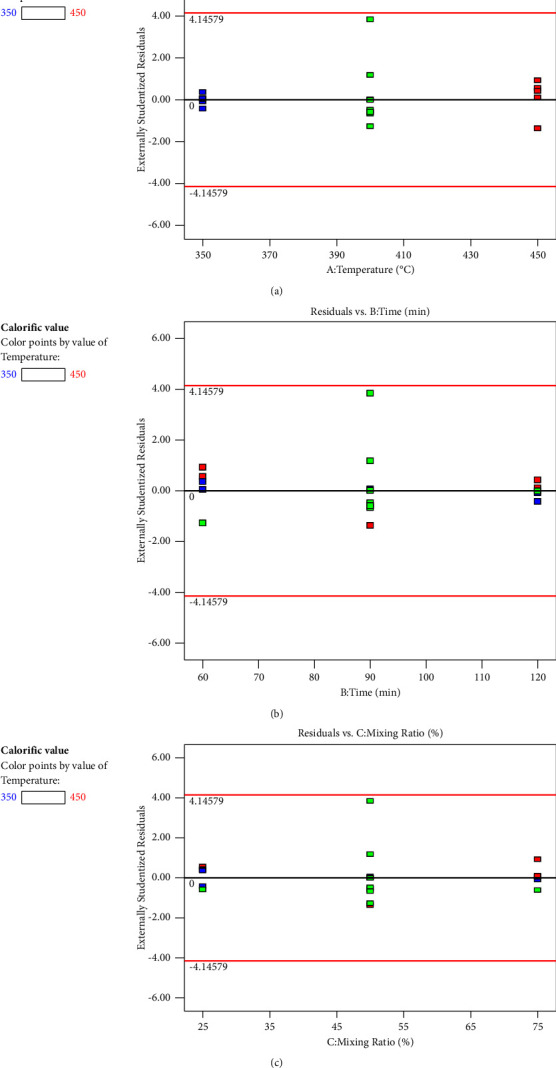
Residuals versus factors' plots: (a) temperature; (b) time; (c) mixing ratio.

Figure 5 displays plots of the residuals against each of the variables (temperature, time, and mixing ratio). It examines if the variation that the model does not account for varies depending on the degree of a factor. If everything is in order, the plot should show a random scatter. The considerable curvature might point to a consistent contribution from the independent element that the model does not take into account.

### 3.5. Effects of Variable Process Variables on the Caloric Value

#### 3.5.1. Effect of Temperature on Calorific Value

**Figure 6:**
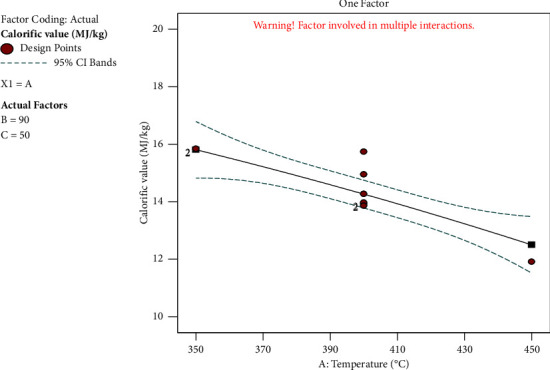
Effect of temperature on calorific value.

Figure 6 depicts how temperature affects the calorific value. The graph shows that the temperature has a negative impact on calorific value. A minimum calorific value of 11.91 MJ/kg was recorded as the temperature climbed up to a maximum of 450°C, indicating that temperature has a significant impact on the carbonation process. Because the maximum calorific value was reached at the lowest temperature, continuing to raise the temperature would reduce it.

#### 3.5.2. Effect of Time on Calorific Value

**Figure 7:**
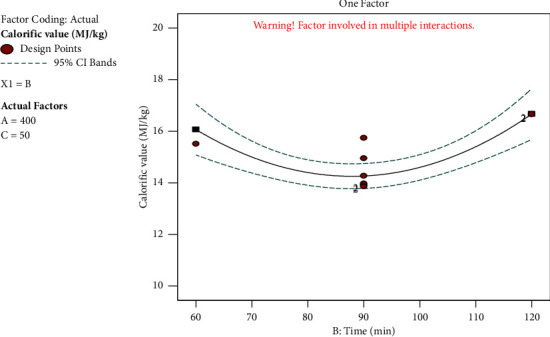
Effect of time on calorific value.

The highest calorific value attained was 19.95 MJ/kg at 350°C and for 60 minutes, while the lowest calorific value was 10.47 MJ/kg at 450°C and for 120 minutes (Figure 7). The outcomes demonstrated that the briquette's maximum calorific value was discovered in the shortest amount of time. This showed that the biomass sample burnt entirely in the shortest amount of time.

#### 3.5.3. Effect of Mixing Ratio on Calorific Value

**Figure 8:**
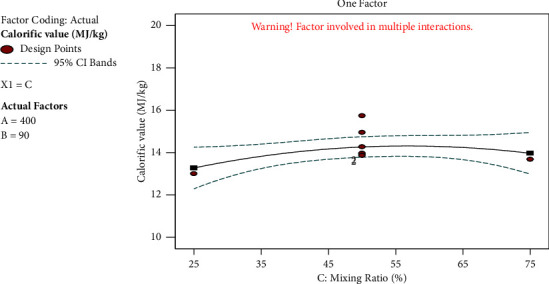
Effects of mixing ratio on calorific value.

Figure 8 illustrates the impact of the mixing ratio on the calorific value. The reaction is influenced favourably by the mixing ratio. A maximum calorific value of 19.95 MJ/kg was attained as the BSG percentage grew to the maximum of 75%, indicating that BSG is essential for the mixing process. The BSG provided the majority of the energy, and when its percentage is increased, it increases the amount of energy it contains. This is because the BSG has a chemical composition that has high fixed carbon content, low ash content, low moisture content, and good volatile matter contents.

### 3.6. Effects of Interaction Variable on Caloric Values

#### 3.6.1. Temperature and Time

**Figure 9:**
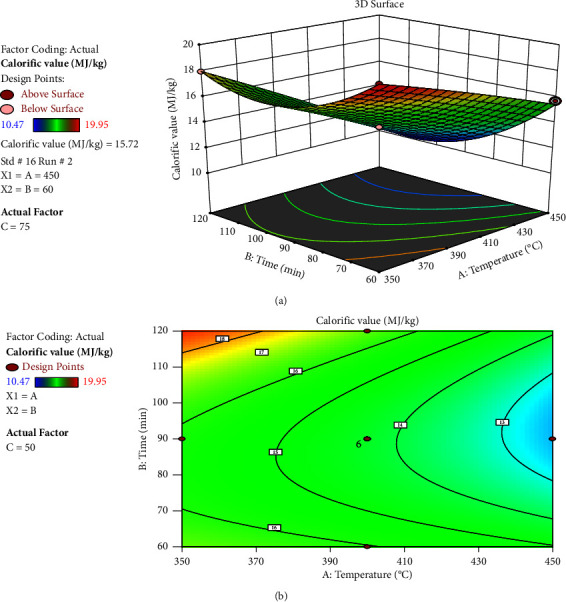
Interaction effect of temperature and time: (a) response surface and (b) contour plot.

Figure 9 depicts the calorific value variation as a function of temperature and time with a constant mixing ratio of 50%. When the time increased from 60 minutes to roughly 83.37 minutes, the calorific value was dropping and then continued to grow gradually until it reached 120 minutes. As the temperature went from 350 degrees Celsius to 450 degrees Celsius, the calorific value marginally reduced. As a result, it is clear from this that time has a greater impact on the carbonization process than temperature. According to the study's findings, time has a greater interaction impact on calorific value than temperature.

#### 3.6.2. Temperature and Mixing Ratio

**Figure 10:**
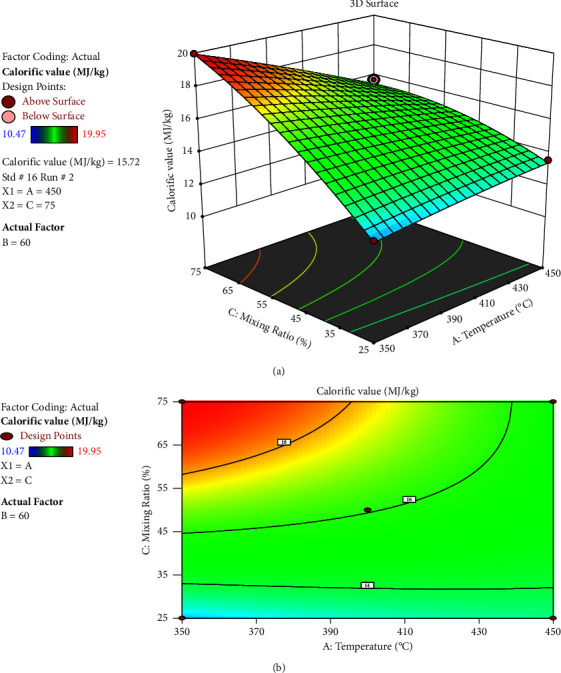
Interaction effect of temperature and mixing ratio: (a) response surface and (b) contour plot.

The calorific value increased when the mixing ratio went from 25 to 75%, while the reaction time remained constant at 90 minutes, despite a modest decrease in calorific value when the temperature rose from 350 to 450 degrees Celsius. According to Figure 10, a high mixing ratio has favoured the reaction and has a stronger interaction effect on calorific value than temperature. As the temperature rose and the mixing ratio shrunk, BSG and BWWS' calorific value dropped.

#### 3.6.3. Time and Mixing Ratio

**Figure 11:**
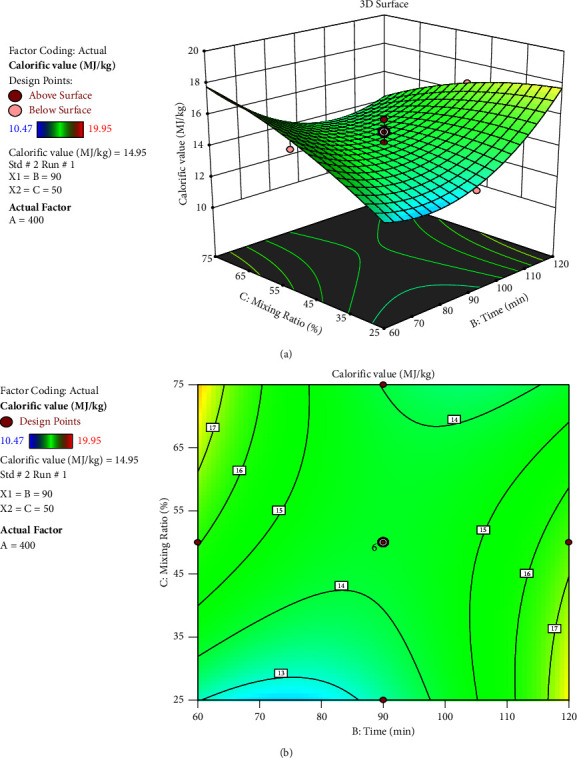
Interaction effect of time and mixing ratio: (a) response surface and (b) contour plot.

According to the plot in Figure 11, as the temperature remained constant at 400°C, the time increased from 60 minutes up to approximately 83.37 minutes, the calorific value decreased before beginning to slightly increase up to 120 minutes, and the calorific value increased as the mixing ratio increased from 25% to 75%. One may deduce from the plot that increased mixing ratios were more favourable for calorific value than they were for reaction times.

### 3.7. Model Validation

**Figure 12:**
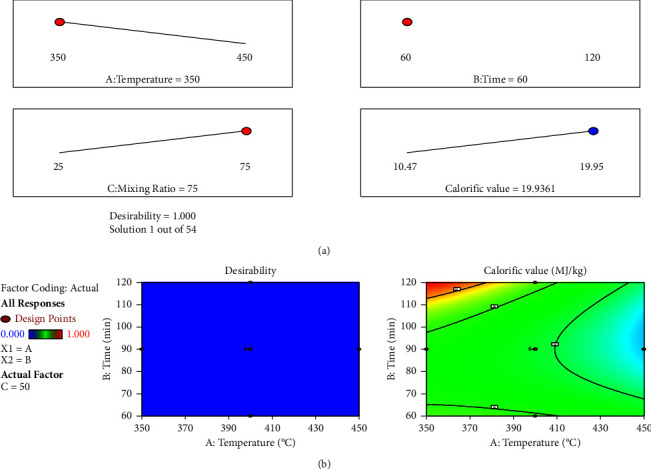
Optimization result with desirability: (a) 2D plot and (b) contour plot.

Triplicate experiments were carried out using the optimised carbonization process parameters in order to validate the optimal condition predicted by the model using the desirability ramp. A calorific value of 19.93 MJ/kg with a desirability value of 0.99 was obtained and shown in Table 9 and Figure 12.

The value of the model's desirability parameter, which is close to unity and has a low error, illustrates how well the model can be applied to the replies. The data predicted from optimization analysis using the desirability function is closely linked to the real or experimental result. The models are appropriate and sufficient to forecast the responses, as evidenced by the error being reached at 0.073%, which is quite minimal and less than 0.5 for the projected and actual values. As a result, this study demonstrates that sawdust briquettes can replace a BSG and BWWS mixture as a substitute energy source for domestic energy sources since they have a high calorific value under ideal carbonation circumstances.

## 4. Conclusion

Due to their high levels of organic matter with a sufficient high heating value, discarded grains and sludge from breweries can be used as a possible resource for the creation of briquettes for renewable fuels. The goal of this study was to examine the caloric value and quality of mixed briquettes made from BSG and BWWS with 20% molasses added as a binding agent to promote agglomeration. The Design-Expert software version 12 was used to conduct 20 experiments on the mixture of BSG: BWWS with different mixing ratios of 25 : 75, 50 : 50, and 75 : 25. In order to make composite briquettes with a maximum caloric content of 19.95 MJ/kg, the ideal conditions were chosen, which included a temperature of 350°C, a production period of 60 min, and a 75% BSG mixing ratio. For mixed carbonized briquettes, the calorific value of densified at 100 bar was 22.55 MJ/kg, but for mixed noncarbonized briquettes, it was 22.13 MJ/kg. The outcome showed that carbonising the combined BSG and BWWS boosted the calorific value to be equivalent with sawdust briquettes of 22.88 MJ/kg. The quadratic model was the response surface, according to the findings of the ANOVA study. *A*, AB, AC, BC, and *B*^2^ are important model terms in this instance. Model terms are not significant if the value is higher than 0.1000. *B*, *C*, *A*^2^, and *C*^2^ are therefore not important model terms. *R*^2^ was 0.9607, which meant that 96.07% of the calorific value correlation between experimental and anticipated values had been validated. The quadratic model would fit the data more effectively under the experimental conditions the greater the value of *R*^2^. The findings can be compared to sawdust briquettes, the subject of several studies and development. In light of their potential to address issues with waste disposal, energy insufficiencies, and air pollution, the study came to the conclusion that using brewery spent grain and BWWS as alternative energy sources is appropriate. Based on the standard ISO/TS 17225 8 : 2016 solid biofuels, the produced briquette from BSG and BWWS using sawdust as alternative energy sources meets those requirements for energy source.

## Figures and Tables

**Table 1:** The full factorial experimental design of briquette production with the three factors and three levels.

Variable	Minimum (−1)	Center point (0)	Maximum (+1)
Temperature (°C)	350	400	450
Time (min)	60	90	120
Mixing ratio of BSG: BWWS (%)	25	50	75

**Table 2:** The Proximate analysis results of BSG, BWWS, and sawdust.

Raw sample	Moisture content (%)	Volatile matter content (%)	Ash content (%)	Fixed carbon	Calorific value (MJ/kg)
BSG	5.11	74.92	4.92	15.05	20.20
BWWS	5.83	53.03	20	3.05	13.80
Sawdust	6.93	75.92	1.90	15.25	20.28
Molasses	24	60.2	8.7	7.1	9.80

BSG is brewery spent grain, and BWWS is brewery wastewater sludge.

**Table 3:** Ultimate analyses of BSG, BWWS, and sawdust.

Raw sample	C (%)	H (%)	N (%)	O (%)
BSG	49.5	7.0	1.7	41.8
BWWS	16.3	2.7	1.9	79.1
Sawdust	43.8	6.2	0.8	49.2

**Table 4:** Experimental design and response results.

Run	Temperature (°C)	Time (min)	Mixing ratio (%)	Calorific value (MJ/kg)
1	400	90	50	14.95
2	450	60	75	15.72
3	400	90	50	14.27
4	400	90	50	13.86
5	350	90	50	15.84
6	350	60	75	19.95
7	450	60	25	13.56
8	400	90	75	13.68
9	350	120	75	17.97
10	350	120	25	18.54
11	450	120	75	10.47
12	450	120	25	16.66
13	400	90	50	15.74
14	400	90	50	13.92
15	400	90	25	13.00
16	400	60	50	15.51
17	450	90	50	11.91
18	350	60	25	12.56
19	400	120	50	16.66
20	400	90	50	13.97

**Table 5:** Optimization conditions of all factors.

Name	Goal	Lower limit	Upper limit	Lower weight	Upper weight	Importance
Temperature	Minimize	350	450	1	1	3
Time	Is in range	60	120	1	1	3
The mixing ratio of BSG: BWWS	Maximize	25	75	1	1	3
Calorific value	Maximize	10.47	19.95	1	1	3

**Table 6:** Physical properties of optimum mixed carbonized sample and briquettes.

Sample	Density (g/cm³)	Calorific value (MJ/kg)
Optimum mixed carbonized sample	—	19.95
Mixed carbonized briquette	1.247	22.55
Mixed noncarbonized briquette	1.118	22.13
Sawdust briquette	0.888	22.88
Safana [36]		10–35
Abu Bakar and Baidurah [37]		21.65-25.18

**Table 7:** Proximate analysis of mixed noncarbonized and carbonized briquette and sawdust briquette.

Sample	Moisture content (%)	Ash content (%)	Volatile matter (%)	Fixed carbon (%)
Mixed noncarbonized briquette	5.37	8.69	63.24	10.53
Mixed carbonized briquette	2.92	10.86	53.49	13.38
Sawdust briquette	6.93	1.90	75.92	15.25
Safana [36]	6–14	<4%	50–90	9–25
Abu Bakar and Baidurah [37]	3.15–4.54	—	—	—

**Table 8:** Fit statistics.

Std. dev.	150.67
Mean	3567.74
C.V. (%)	4.22
PRESS	5.910*E* + 05
*R* ^2^	0.9607
Adjusted *R*^2^	0.9253
Predicted *R*^2^	0.8976
Adeq precision	21.2830

**Table 9:** Model validation of calorific value.

Response	Desirability	Temperature	Time	Mixing ratio	Predicted	Experimental	Error
Calorific value	0.99	350	60	75	19.93	19.95	0.073

## Data Availability

All the data are included in the manuscript.
